# Supplementary material for: Identification and functional analysis of non-coding regulatory small RNA FenSr3 in Bacillus amyloliquefaciens LPB-18
Source: PeerJ. 2023 May 15;11:e15236. doi: 10.7717/peerj.15236 (PMC10194069; doi:10.7717/peerj.15236)
Supplement: Supplemental Information 4 [file peerj-11-15236-s004.zip › KO/CK-vs-T1_map/map05414.html]

KEGG PATHWAY: Dilated cardiomyopathy (DCM) - Reference pathway


|  |  |
| --- | --- |
| **Dilated cardiomyopathy (DCM) - Reference pathway** |  |

[
Pathway menu
| Organism menu
| Pathway entry
| Show description
| User data mapping
]

Dilated cardiomyopathy (DCM) is a heart muscle disease characterised by dilation and impaired contraction of the left or both ventricles that results in progressive heart failure and sudden cardiac death from ventricular arrhythmia. Genetically inherited forms of DCM ("familial" DCM) have been identified in 25-35% of patients presenting with this disease, and the inherited gene defects are an important cause of "familial" DCM. The pathophysiology may be separated into two categories: defects in force generation and defects in force transmission. In cases where an underlying pathology cannot be identified, the patient is diagnosed with an "idiopathic" DCM. Current hypotheses regarding causes of "idiopathic" DCM focus on myocarditis induced by enterovirus and subsequent autoimmune myocardium impairments. Antibodies to the beta1-adrenergic receptor (beta1AR), which are detected in a substantial number of patients with "idiopathic" DCM, may increase the concentration of intracellular cAMP and intracellular Ca2+, a condition often leading to a transient hyper-performance of the heart followed by depressed heart function and heart failure.

|  |  |  |
| --- | --- | --- |
| Reference pathway | 184% 150% 122% 100% 82% 67% 55% | 图片下载 |
